# Enhancing alfalfa breeding through genomic prediction with exotic germplasm resources

**DOI:** 10.1186/s12870-026-09507-5

**Published:** 2026-07-18

**Authors:** Shufen Chen, Meng Lin, Neal W. Tilhou, Bhoja R. Basnet, Dongyan Zhao, Craig T. Beil, Heathcliffe Riday, Moira J. Sheehan

**Affiliations:** 1 Breeding Insight, Cornell University, 525 Tower Rd, Ithaca, NY 14853 USA; 2 Breeding Insight, Institute of Food and Agricultural Sciences, University of Florida, PO Box 110695, Gainesville, FL 32611 USA; 3 US Dairy Forage Research Center, USDA-ARS, 1925 Linden Dr, Madison, WI 53706 USA

**Keywords:** Alfalfa, Genomic prediction, Exotic germplasm, Perennial cover crop, DArTag genotyping

## Abstract

**Background:**

The adoption of molecular breeding methods and genomic selection in alfalfa breeding has lagged behind that of major crops such as corn and soybean. Discovery and introgression of native traits into new cultivars desired by growers requires breeders and pre-breeders to leverage genomic information from exotic germplasm sources into their breeding pipelines.

**Results:**

We investigated the effects on genomic predictive ability when the composition and proportions of the germplasm used in the training and test sets vary in genomic prediction schemes. We used a set of 780 alfalfa samples collected from different geographic origins (CASIA, EURO, OTTM, and SIBR) plus U.S. breeding materials (Check) evaluated in a multi-year field trial for growth habit (GH), plant height (HGT), and plant vigor (VIG), and genotyped using the alfalfa 3 K DArTag panel. The genotyping results revealed distinct patterns of linkage disequilibrium across the five germplasm subgroups. Genomic prediction across the entire population outperformed predictions within individual subgroups. Further optimization was achieved by systematically varying the proportion of samples from each of the five subgroups in the training set (10–90%). Additionally, when increasing the number of test subgroup samples to the training set, the increased genetic relatedness between the training and test sets could be a major driver of improved predictive performance.

**Conclusions:**

Overall, these results indicate that successful genomic prediction in alfalfa requires not only expanding training set sizes but also strategically incorporating highly diverse germplasm and evaluating newly introduced base populations to maximize predictive ability.

**Supplementary Information:**

The online version contains supplementary material available at https://doi.org/10.1186/s12870-026-09507-5.

## Background

Alfalfa (*Medicago sativa* L.) is a deep-rooted perennial legume that plays a crucial role in sustainable agriculture due to its ability to fix atmospheric nitrogen through a symbiotic relationship with the soil bacterium *Sinorhizobium meliloti* [1]. This nitrogen-fixing capability reduces reliance on exogenously applied nitrogen fertilizers, thereby enhancing the sustainability and environmental resilience of farming systems. Alfalfa is the most important forage legume globally for its high biomass yield, high nitrogen-fixing capacity, nutritional value, and broad adaptability in animal feeding [2, 3]. The genetic diversity within medic species, spanning diploid wild relatives (2n = 2x = 16) and autotetraploid cultivated varieties (2n = 4x = 32), provides a critical reservoir for alfalfa breeding programs [1, 4, 5].

Alfalfa breeding objectives primarily focus on enhancing agronomic traits, including forage yield, regrowth, digestibility, and resistance to biotic and abiotic stresses [1]. Several key traits, including growth habit, biomass yield, height, and regrowth after cutting, are crucial for selecting improved cultivars. Traditional breeding techniques in alfalfa, mainly employing phenotypic recurrent selection, have played a significant role in genetic improvement [1]. However, most key traits are influenced by both quantitative genetics and environmental factors, making their improvement through traditional breeding more challenging. In recent years, significant advancements in alfalfa breeding have been achieved through the integration of cutting-edge molecular breeding technologies, including genomic prediction (GP) [6]. The advantages of GP in plant breeding programs include, but are not limited to, capturing both major- and minor-effect loci, modeling complex trait relationships, and accelerating the breeding cycle. Improving the predictive ability of GP is crucial for the successful integration of genomic selection into breeding programs. Several studies on GP have been conducted in alfalfa [7–9], and other crop species such as wheat [10, 11], maize [12, 13], and rice [14, 15], showcasing improvements of predictive ability by using different statistical methods, experimental designs, and sampling schemes. Understanding population structure within breeding programs is essential for optimizing genomic prediction models. Due to differences in allele frequencies, subpopulations can exhibit substantial genetic diversity, which affects the prediction accuracy of GP [16].

Genetic markers have proven valuable for assessing genetic diversity and enhancing genetic improvement in breeding populations [17]. Recently, a Diversity Array Technology targeted genotyping platform (DArTag) was developed for alfalfa, featuring 3 K markers distributed across the alfalfa genome [18]. Because the DArTag platform is a targeted amplicon marker system, the panel not only captures the target single-nucleotide polymorphism (SNP) at each locus but also detects closely linked off-target SNPs in the short-read amplicons produced. The 3 K DArTag panel has enabled researchers to study genetic diversity, create linkage maps, identify quantitative trait loci (QTL), perform genome-wide association studies (GWAS), and implement genomic prediction in alfalfa breeding populations [8, 19–21], highlighting its significant impact on alfalfa genetic research and breeding.

Increasing the genetic diversity of breeding parents by using exotic germplasm collections in crop breeding can reduce the risk of crop vulnerability stemming from historical genetic bottlenecks. In alfalfa, public breeders have implemented a long-term base-broadening program to develop novel germplasm populations for integration into commercial alfalfa cultivar breeding programs [20]. As part of this effort, four geographically based highly diverse germplasm pools were selected to serve as subgroups: Central Asia (CASIA), northeastern Europe (EURO), the Balkans-Turkey-Black Sea region (Ottoman; OTTM), and Siberia/Mongolia (SIBR) [22]. However, the divergence between these collections may pose a challenge for genomic prediction. To establish effective genomic prediction workflows for alfalfa breeding using highly diverse germplasm, it is crucial to explore genetic relationships within and among subgroups to optimize both training and test populations. By strategically selecting genetically diverse yet related training and test populations, breeders can improve predictive ability and enhance the applicability of genomic selection. The objectives of this study were to (1) characterize the genetic diversity for exotic germplasm collections, and (2) evaluate the effects of population structure, population composition, genetic relatedness between the training and test sets, and the size of the training set on GP ability using different prediction schemes. Ultimately, these efforts contribute to the development of effective genomic prediction workflows for integrating highly diverse germplasm resources into breeding programs for cultivated alfalfa.

## Methods

### Plant materials and experimental design

Four polycrossed populations were used in this study. A total of 85 Plant Introduction (PI) accessions (Supplemental Table S1) were selected from 150 PI accessions from the USDA National Plant Germplasm System (NPGS) based on their general agronomic adaptation performance to create the polycrossed populations [22]. Furthermore, the 150 PI accessions used as the base germplasm were all designated as *Medicago sativa* subsp. *sativa* cultivars or landraces by NPGS, but visual observation in the field indicated some populations had individuals with variegated flowers, suggesting at a minimum some subsp. *varia* present in some accessions. These 85 PIs were organized into four groups based on their Eurasian geographic origins: Central Asia (CASIA; 23 PIs), northeastern Europe (EURO; 23 PIs), Balkans-Turkey-Black Sea (Ottoman; OTTM; 22 PIs), and Siberia/Mongolia (SIBR; 17 PIs). One to nine plants per PI were sown as parents to make the polycrosses.

Polycrosses were conducted within each group (CASIA, EURO, OTTM, and SIBR) in insect-isolating pollination enclosures at the former Pioneer Hi-Bred International breeding station in Connell, Washington, during the summer of 2015. Seeds from each of the four polycrosses were harvested on a half-sib basis. Seeds from each half-sib family within the four groups were bulked to form the four polycrossed populations, which are referred to as subgroups later in the text. Additionally, four U.S. commercial lines, including ‘55H94’ (Pioneer Hi-Bred Int.; [23, 24], ‘Genoa’ (Forage Genetics Int.; [25–27], ‘HybriForce-4400’ (Dairyland Seed Co.; [28, 29], and ‘Vernal’ [30] were used to represent high-yielding elite germplasm adapted to the U.S. climate. These commercial lines formed the Check subgroup.

### Phenotypic data collection

Alfalfa seedlings were started in a greenhouse and later transplanted directly into a fine fescue turf (*Festuca rubra* 40%, *Festuca brevipila* 50%, and *Festuca ovina* 10%) in a Richwood silt loam soil (fine-silty, mixed, superactive, mesic Typic Argiudolls) at the U.S. Dairy Forage Research Dairy Farm at Prairie du Sac, Wisconsin (43°20’54.6"N 89°45’24.5"W) on June 13, 2016. The five subgroups mentioned above (CASIA, EURO, OTTM, SIBR, and Check) were transplanted into six-plant plots with a spacing of 60 cm apart in transplant rows, 90 cm between plots within transplant rows, and 75 cm between transplant rows. There were 43 plots of CASIA, EURO, OTTM, and SIBR each, and 20 plots for Check (five plots of ‘55H94’, ‘Genoa’, ‘HybriForce-3400’, and ‘Vernal’ each). A total of 192 plots were organized into four equal-sized blocks (48 plots/block) containing four sub-blocks of 12 plots each. The five subgroups were distributed as evenly as possible across the sub-blocks within blocks.

The spaced plant plots were typically mowed three times per growing season from 2016 to 2019. Mowing dates and phenotyping dates are summarized in Supplemental Figure S1. Plants were visually evaluated for biomass yield, termed vigor, usually about 30 days after a mowing event, except that the first visual evaluation was performed one month before the first cut in 2017. Visual vigor scores (a zero to nine scale, where zero represents death and nine represents the highest vigor) that has been validated in perennial forages [31] and routinely used in breeding programs [32–34] as a resource efficient surrogate for biomass yield, were taken three or four times in 2016, 2017, and 2018 as a low-cost proxy for the total above-ground forage yield per plant (Supplemental Figure S1). A minimum of two vigor scores was required for an individual plant to be considered successfully established after transplantation. Any individual plants that failed to establish after transplanting were omitted from the dataset. To control for variation between individual phenotypes scoring vigor, single scores within each 48-plot block within an observation time point were range transformed using the formula below:

$$\:{Transformed\:vigor}_{ij}=9\:\times\:\:\frac{{Vigor}_{ij}}{{Vigor}_{j}^{99}}$$

where $\:{Vigor}_{j}^{99}$ is the 99^th^ percentile vigor score in the *j*^th^ observation set. The post-transformed score (i.e., VIG) ranged from zero to nine, with occasionally a few values exceeding nine.

Plant height (HGT) was measured per plant in centimeters at five time points across 2017, 2018, and 2019. Plant growth habit (GH) was a visual score used to quantify the gradations from prostrate to upright plant habit (similar to [35]) and was evaluated four times in 2017 (Supplemental Figure S1). Plant growth habit scores ranged from one to nine, with one being most decumbent with the most upright stems growing at a 10° angle or less off the ground, five being intermediate with the most decumbent stems growing at a 50° angle to the ground, and nine being the most upright with all stems growing perpendicular to the ground at a 90° angle.

### Phenotypic data analysis

A single-stage, fully efficient mixed model approach was used to analyze the phenotypic data. For each trait under estimation, three models with different variance structures for random and residual terms were evaluated using the R-package ASReml v4.2 [36]. In each of the three tested models, the observed response for each plot was modeled as a function of an overall mean, fixed effects for cut and plant, random effects for field layout (range & row) across cuts, and a residual error term. The three tested models differed in how range and row effects were modeled: (1) estimate the range and row effects independently, (2) estimate both individual and interaction effects between range and row, and (3) estimate the spatial random effect using a two-dimensional first-order autoregressive (AR1 × AR1) structure across range and row positions as described in [21]. The best model for each trait was chosen based on the lowest Bayesian Information Criterion (BIC) and Akaike Information Criterion (AIC) values [37] (Supplemental Table S2), while maintaining the same fixed effect factors across all tested models. Best linear unbiased estimates (BLUEs) (Supplemental Table S3) for each trait were estimated across all cuts within a year (GH, VIG_2016, VIG_2017, and VIG_2018) or across several years (HGT, VIG_2016&17, VIG) using the selected models. Although the experiment lacked true replicates within a single cut, combining phenotype assessments across cuts or years as repeated measurements allowed estimation of genetic effects and the parsing out of environmental and field spatial variance. The phenotypic correlations among traits, based on BLUE values, were assessed by Pearson’s correlation and visualized using the pairs.panels function in R-package psych v2.5.6 [38].

To estimate genotypic variance, a separate mixed model analysis was carried out using plant as random factor, and generalized heritability was estimated based on method proposed by [39] as below:

$$\:{h}^{2}=1-\frac{{A}_{tt}}{{2\sigma\:}_{g}^{2}}$$

Where, $\:{h}^{2}$= generalized heritability proposed by Cullis et al. (2006) [39] ;$\:{\sigma\:}_{g}^{2}$ = genetic variance; $\:{A}_{tt}$ = average pairwise prediction-error variance (PEV) of pairwise differences between genotypes:

$$\:{A}_{tt}=E\left[Var\left({\widehat{g}}_{i}-{\widehat{g}}_{j}\right)\right]\approx\:2*\overline{PEV}$$

where $\:{\widehat{g}}_{i}$ and $\:{\widehat{g}}_{j}$ model based are predicted phenotypic values for genotype *i* and *j.*

### Genotypic data

A total of 798 samples were genotyped using the alfalfa 3 K DArTag marker panel (Alfalfa_DArTag_BI_Cornell_University (1.0)). The genotyping results presented in the “missing allele discovery count” (MADC) file by DArT encompass all microhaplotypes (81-bp amplicon sequences) identified by a consensus of amplicons at each of the 3 K marker loci. These microhaplotypes included reference and alternative microhaplotypes containing Reference (Ref) and Alternative (Alt) bases at the target SNP sites, as well as RefMatch (target variant site matches reference) and AltMatch (target variant site matches alternative) microhaplotypes containing off-target SNPs in addition to the reference and alternative allele at the target SNP sites. To ensure accurate analyses within and between projects, filtering was applied at the microhaplotype level using the following steps: (1) all the RefMatch and AltMatch microhaplotypes were filtered, requiring data in at least 10 samples, each with a minimum of 2 reads; (2) retained microhaplotypes were then aligned to the 81 bp Ref and Alt sequences of markers using NCBI BLAST v2.11.0+ [40]. Only those with ≥ 90% identity over ≥ 90 coverage were kept; (3) for DArTag loci with > 10 microhaplotypes (potentially indicating paralogous sequence amplification), only Ref and Alt alleles were retained. Subsequently, filtering was applied at both the sample and loci level. A total of 18 samples were removed due to their high missing rate, ranging from 60% to 100%, calculated as the number of missing loci (loci with < 10 total reads per sample) over the 3,000 total loci. After removing the samples, 2,931 (2.3% missing rate) marker loci were retained with 10,994 microhaplotypes (≥ 10 reads in > 20% of samples) across 780 samples. To identify all variants, multiple sequence alignment was performed to extract variants across 10,994 microhaplotypes, including Ref, Alt, RefMatch, and AltMatch sequences, using the R-package msa v1.38.0 [41]. A total of 7,615 SNPs, including both target and off-target SNPs, were extracted from 10,994 microhaplotypes of the 2,931 amplified marker loci across 780 samples (n_CASIA_=155, n_EURO_=180, n_OTTM_=178, n_SIBR_=176, and n_Check_=91). Read depths of each variant site were calculated by collapsing read depths of all microhaplotypes containing that genomic variant.

### Dosage calling using read counts

Genotype dosage calling was performed for 7,615 SNPs from 780 samples using the “multidog” function in the R-package updog v2.1.5 [42]. Dosages were called using the “norm” model with the ploidy level set to four. After dosage calling, ensuring high confidence genotype calls while minimizing inclusion of poorly fitted or low information markers, 3,756 SNPs were retained based on the following criteria: the estimated allele bias (0.05 < bias < 2) to retain SNPs with balance allelic representation, the estimated proportion of individuals misclassified (prop_mis < 0.1) to retain SNPs with low misclassification rates, the estimated overdispersion parameter (od < 0.05) to retain markers consistent with the expected read count distribution, and minor allele frequency (MAF ≥ 0.03) to remove rare SNPs.

### Population structure analysis

A kinship matrix was computed for 3,756 SNPs from 780 samples using the set.K function in the R-package GWASpoly v2.13 [43]. Then, principal component analysis (PCA) was conducted by performing eigen decomposition of the kinship matrix using the eigen function in R [44]. Linkage disequilibrium (LD) decay for the entire population and each of the five subgroups was calculated using 3,756 SNPs with the LD.plot function of the R-package GWASpoly v2.13 [43], and plotted using the ggplot2 v3.5.1 [45]. The pairwise genetic differentiation (*F*_*ST*_) and Nei’s genetic distance (*D*_*A*_) between pairs of subgroups were evaluated using the “stamppFst” and “stamppNeisD” functions, respectively, in the R-package StAMPP v1.6.3 [46]. The results were visualized using a heatmap generated by the R-package ComplexHeatmap v2.24.1 [47].

### Genomic prediction model

Genomic prediction for each trait was performed using the mixed.solve function in the R-package rrBLUP v4.6.3 [48] by following the model:

$$\:y=\mu\:+Zu+\varepsilon\:$$

Where * y* is the vector of BLUEs for a given trait; $\:\mu\:$ is the vector of overall mean as a fixed effect; * u* is a vector of random marker effects with * u*~*N*(0, $\:{I\sigma\:}_{u}^{2}$) where $\:{\sigma\:}_{u}^{2}$ is the additive genetic variance; Z is an incidence matrix for SNP genotypes; $\:\varepsilon\:$ is the random residual effect, $\:\varepsilon\:$ ~*N*(0, $\:{I\sigma\:}_{\varepsilon\:}^{2}$) where $\:{\sigma\:}_{\varepsilon\:}^{2}$ is the residual variance, *I* is the identity matrix with a size the same as the number of used samples.

### Genomic prediction scenarios

To explore the effects of population structure, population composition, genetic relatedness between the training and test sets, and the size of the training set, and temporal variation on predictive ability, multiple genomic prediction scenarios were developed to reflect the diverse situations breeders may encounter when implementing genomic selection. These scenarios aimed to provide insights into the optimal design of training populations for alfalfa genomic prediction.

#### Genomic prediction within each of the five subgroups versus across the entire population

Genomic prediction was performed within each of the five subgroups or across the entire population using a five-fold cross-validation design with fifty iterations. In each fold, the dataset was randomly partitioned into five equal subsets, with one subset designated as the test set and the remaining four subsets as the training set. Predictive ability for each trait in each subgroup or the entire population was represented as the Pearson correlation between the predicted phenotypic values and the BLUEs.

#### Genomic prediction with equal-proportion sampling from subgroups

**Fig. 1:**
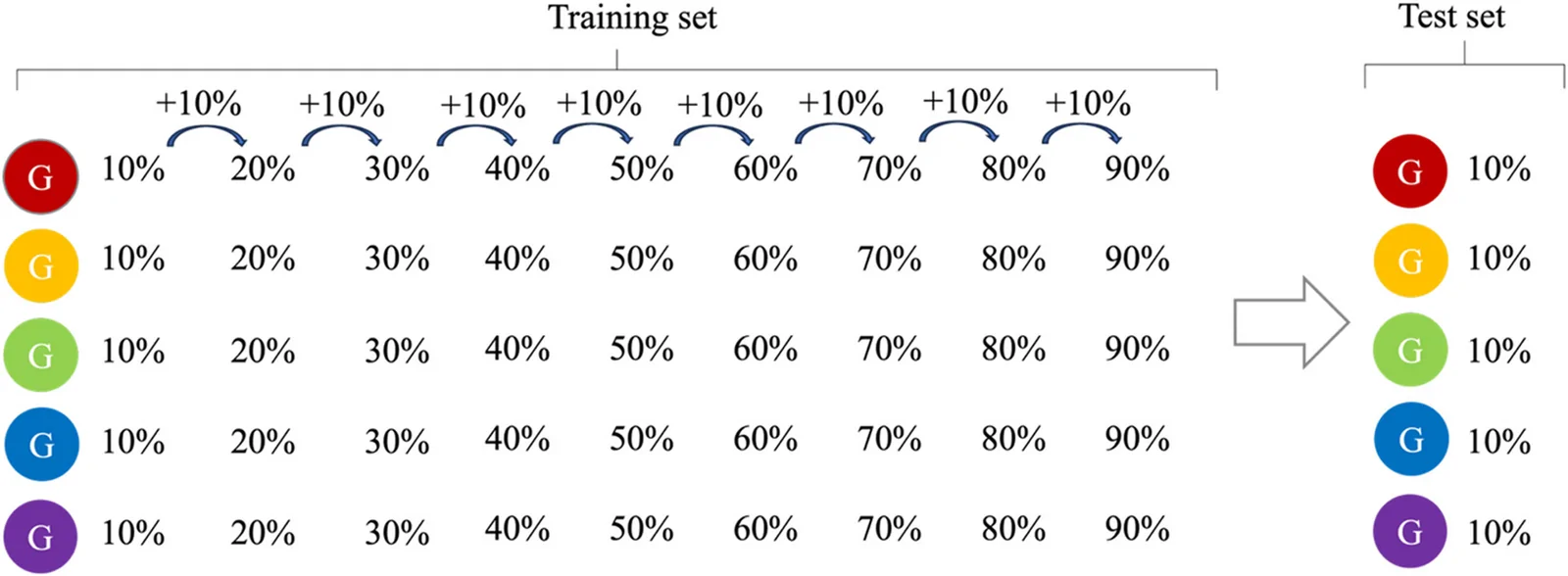
Genomic prediction scenarios. 10% of samples from each of the five groups (G) was used as the test set, while the training set was designed to include the same proportion (from a randomly selected 10% to 90%, in 10% increments) of remaining samples from each of the five groups. G with different colors represents the different subgroups

To investigate the effect of different sample proportions from each of the five subgroups in the training set required to achieve comparable predictive performance, genomic prediction (Fig. 1) was performed using a hold-out validation design with 50 iterations. For each iteration, a randomly selected 10% of samples from each of the five groups was used as the test set, while the training set was designed to include the same proportion (from a randomly selected 10% to 90%, in 10% increments) of remaining samples from each of the five groups. Predictive ability for each trait was measured at each proportion by the Pearson correlation between the predicted phenotypic values and the BLUEs.

#### Genomic prediction with increasing samples from training subgroups versus the test subgroup to the training set

**Fig. 2:**
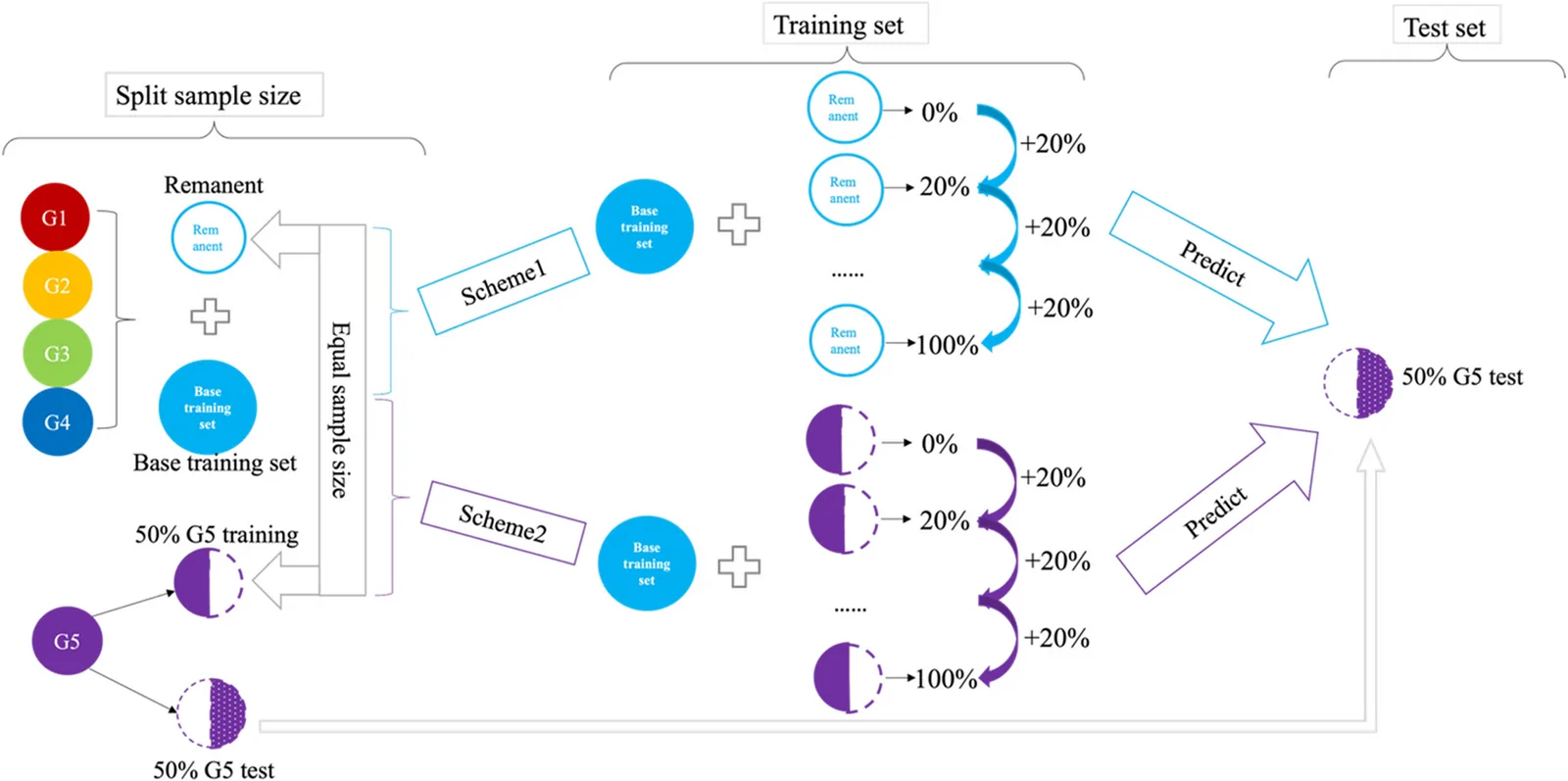
Genomic prediction scenarios. 50% of samples from the fifth subgroup (G5), which served as the test subgroup were used as the test set. Additionally, samples from the other four training subgroups, after excluding a subset equal in size to the remaining 50% of the test subgroup, were used as the base training set, while the two schemes differed only in the composition of the additional samples added to the training set. Scheme1: The excluded samples from the other four training subgroups were added to the training set with increasing proportions (0%–100%, in 20% increments). Scheme2: The remaining 50% of the test subgroup, equal in size to the excluded samples in Scheme1, was added to the training set with increasing proportions (0%–100%, in 20% increments). G with different colors represents the different subgroups

The two schemes (Fig. 2) were developed to explore the effect of the genetic relationship between the training and test sets on predictive ability. Genomic prediction was performed using a hold-out validation design with fifty iterations in these two schemes. Predictive ability for each trait in each subgroup was quantified as the Pearson correlation between predicted phenotypic values and BLUEs. For each iteration, the test set comprised 50% of the samples from the fifth subgroup (G5), which served as the test subgroup, ensuring that each subgroup was used in turn as the test subgroup. Additionally, samples from the other four training subgroups, after excluding a subset equal in size to the remaining 50% of the test subgroup, were used as the base training set, while the two schemes differed only in the composition of the additional samples added to the training set. 

Specifically, in Scheme1, samples from the test subgroup were not included in the training set. The excluded samples from the other four training subgroups were added to the training set with increasing proportions (0%–100%, in 20% increments). In Scheme 2, samples from the test subgroup were included in the training set. The remaining 50% of the test subgroup, equal in size to the excluded samples in Scheme1, was added to the training set with increasing proportions (0%–100%, in 20% increments).

To explore whether changes in predictive ability were also accompanied by changes in genetic relatedness between the test and training sets with increasing proportions of training samples (0%–100%, in 20% increments) under the two schemes, the genetic relatedness was quantified as the mean kinship of test samples to the corresponding training set that was calculated from the overall kinship matrix constructed using all samples (described in Methods section: populations structure analysis) for each proportion.

#### Genomic prediction for plant vigor across different years

To evaluate predictive ability across years, genomic prediction was performed for VIG using BLUEs from 2016, 2017, 2018, and the combined 2016 and 2017 VIG data. Five-fold cross-validation was performed with fifty iterations. Specifically, the 2016 dataset was used to predict phenotypic performance in 2017 and 2018, while the 2017 dataset was also used independently to predict performance in 2018. Additionally, a combined dataset from 2016 to 2017 was used to predict 2018 performance. Predictive ability was assessed by the Pearson correlation between the predicted phenotypic values from the training year and the BLUEs from the target year. Furthermore, SNP marker effects were estimated using rrBLUP based on the BLUEs for 2016, 2017, 2018, and the combined VIG dataset of 2016 and 2017. Pearson correlation coefficients of SNP marker effects were then calculated to assess the stability of marker effects across years, including comparisons between 2016 and 2017, 2016 and 2018, 2017 and 2018, and between the combined 2016–2017 dataset and 2018.

## Results

### Phenotypic variation and correlation

Phenotypic variation was assessed across the entire population and within each subgroup for GH, HGT, and VIG, as indicated by their BLUEs (Table 1). For the entire population, the BLUEs ranged from 1.7 to 8.6 for GH (mean score = 4.8), 6.4 to 66.5 for HGT (mean = 38.6 cm), and 0.2 to 8.4 for VIG (mean score = 4.0). Although all three traits exhibited broad phenotypic ranges across the entire population, the extent of phenotypic variation differed among subgroups. SIBR consistently displayed the highest CV values across all traits, followed by EURO and CASIA. In contrast, the Check subgroup consistently exhibited the lowest CV values for all three traits. Notably, however, the Check subgroup also showed the highest average VIG value, consistent with its composition of high-yielding commercial cultivars that are well adapted to North American growing conditions. Heritability estimates were 0.259 for GH, 0.652 for HGT, and 0.753 for VIG. However, these estimates are not directly comparable because the traits were evaluated across different time periods and years.

**Table 1:** Summary of range, means, standard deviation (SD), coefficient of variation (CV), and heritability (*h*^*2*^) for phenotypic traits based on best linear unbiased estimates (BLUEs)

Traits	Group	Range	Mean	SD	CV	*h* ^2^
GH	All	1.7~ 8.6	4.8	1.0	20.2%	0.259
	OTTM	3.1 ~ 8.6	5.1	0.8	15.2%	-
	SIBR	2.0 ~ 6.3	4.0	0.9	22.4%	-
	CASIA	1.7 ~ 8.5	5.4	0.9	16.6%	-
	EURO	3.0 ~ 8.3	4.9	0.9	17.9%	-
	Check	3.2 ~ 6.4	4.8	0.7	14.4%	-
HGT	All	6.4~ 66.5	38.6	11.4	29.6%	0.652
	OTTM	11.4 ~ 66.5	47.3	9.8	20.6%	-
	SIBR	7.4 ~ 45.4	27.2	7.0	25.8%	-
	CASIA	6.4 ~ 59.4	39.8	9.5	24.0%	-
	EURO	10.9 ~ 56.5	36.7	9.2	25.0%	-
	Check	15.1 ~ 61.6	45.5	7.9	17.4%	-
VIG	All	0.2~ 8.4	4.0	1.6	40.3%	0.753
	OTTM	0.3 ~ 8.3	4.7	1.6	34.4%	-
	SIBR	0.3 ~ 7.4	3.6	1.6	43.9%	-
	CASIA	0.3 ~ 8.4	3.5	1.5	42.8%	-
	EURO	0.2 ~ 7.4	3.9	1.4	37.3%	-
	Check	0.2 ~ 7.9	5.3	1.4	26.7%	-

The correlations between traits varied (Supplemental Figure S2). VIG across years exhibited consistently strong positive correlations with VIG by individual years (*r* = 0.87 for 2016; *r* = 0.93 for 2017; *r* = 0.76 for 2018) as well as with the combined 2016–2017 dataset (*r* = 0.98). Similarly, strong positive correlations were also observed among adjacent years (0.63 ~ 0.94), whereas relatively weaker correlations were observed for more temporally distant comparisons (*r* = 0.39 for 2016 vs. 2018; *r* = 0.58 for combined 2016–2017 vs. 2018). HGT showed a high positive correlation with VIG across years (*r* = 0.68), also reflected moderate to high correlations with VIG by year 2016 (*r* = 0.53), 2017 (*r* = 0.72), 2018 (*r* = 0.39), and combined 2016 and 2017 (*r* = 0.70). In contrast, GH showed weak positive and negative correlations with HGT (*r* = 0.27) and VIG (*r* = − 0.23), respectively, with consistently weak negative correlations with VIG by year, including 2016 (*r* = − 0.12), 2017 (*r* = − 0.18), 2018 (*r* = − 0.28), and the combined 2016–2017 dataset (*r* = − 0.18).

### Population structure, linkage disequilibrium, and genetic differentiation

Principal component analysis (PCA) was performed to assess the genetic diversity among subgroups. The first three PCs explained 4.9% (PC1), 2.0% (PC2), and 1.6% (PC3) of the total genetic variance in the population (Supplemental Figure S3). Individuals from the same subgroups were generally clustered together. CASIA, EURO, and SIBR subgroups are more distinct from the other subgroups, while EURO, OTTM, and Check samples partially overlap. The overall pattern from PCA aligned with the geographic origins of the subpopulations and indicated that these five subgroups had distinct genetic backgrounds.

Estimates of linkage disequilibrium (LD) decay distances, calculated at the *r*^*2*^ threshold of 0.1, varied across the entire population and the five subgroups **(**Supplemental Figure S4). The LD decay distance was approximately 0.50 Mb for the entire population (All), while subpopulations showed longer decay distances: 1.02 Mb in OTTM, 1.47 Mb in Check, 1.70 Mb in EURO, 2.12 Mb in CASIA, to 2.36 Mb in SIBR. The reduced LD decay distance observed in the entire population (0.50 Mb) relative to subgroups suggested greater genetic diversity and historical recombination events in the broader genetic pool.

The genetic differentiation pairwise coefficient (*F*_*ST*_) and Nei’s genetic distance (*D*_*A*_) estimates among subgroups were generally low (Supplemental Figure S5). Pairwise (*F*_*ST*_) among subgroups that were significantly different (*p* < 0.001), ranged 0.012 (between OTTM and EURO subgroups) to 0.039 (between SIBR and CASIA subgroups). Similarly, *D*_*A*_ was greatest between the SIBR and CASIA subgroups (0.02) and lowest between OTTM and EURO (0.006).

### Assessment of genomic prediction performance within each of the five subgroups versus across the entire population

**Fig. 3:**
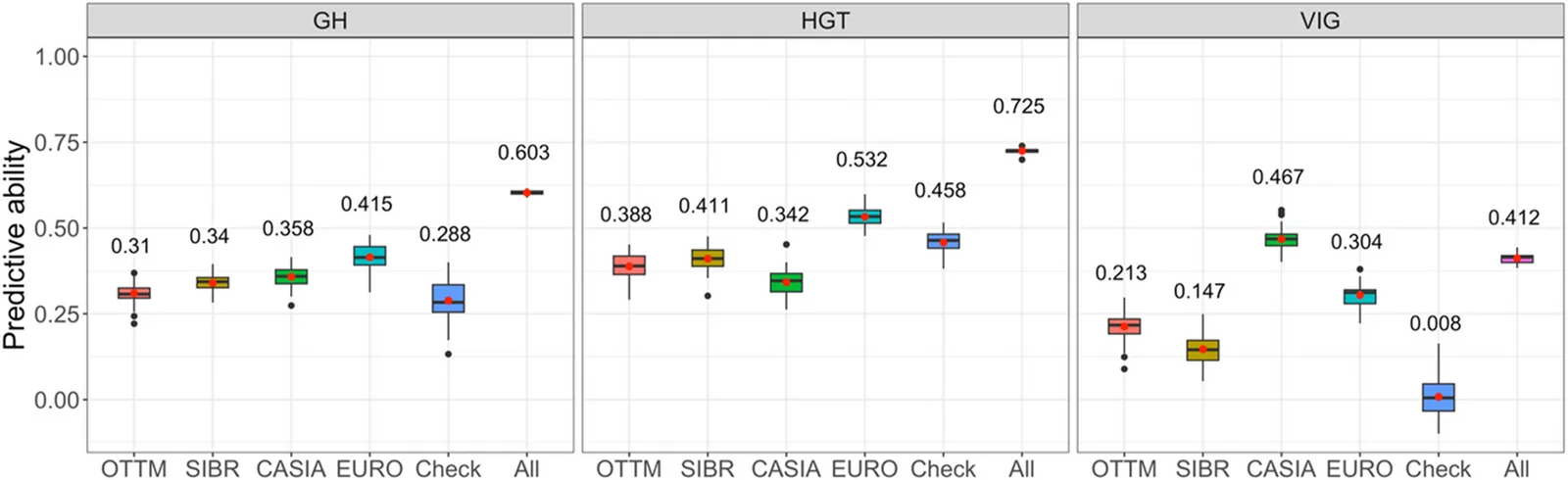
Predictive abilities for traits within each of the five subgroups (OTTM, SIBR, CASIA, EURO, and Check) and across the entire population (All). Red dots represent each group’s mean predictive ability, with specific values displayed above the interquartile-range boxes

Predictive abilities for traits varied among subgroups (Fig. 3; Supplemental Table S4), with average values ranging from 0.288 (Check) to 0.415 (EURO) for GH, 0.342 (CASIA) to 0.532 (EURO) for HGT, and − 0.007 (Check) to 0.467 (CASIA) for VIG. Across the entire population (All), predictive abilities were substantially higher, particularly for GH (0.603) and HGT (0.725), representing improvements of 45% (EURO) to 109% (Check) and 36% (EURO) to 112% (CASIA), respectively, compared to those within subgroups. For VIG, the predictive ability in the entire population (0.412) was slightly lower than that of CASIA but higher than those of other subgroups, with an increase of > 36%.

### Assessment of genomic prediction performance with equal-proportion sampling from subgroups

**Fig. 4:**
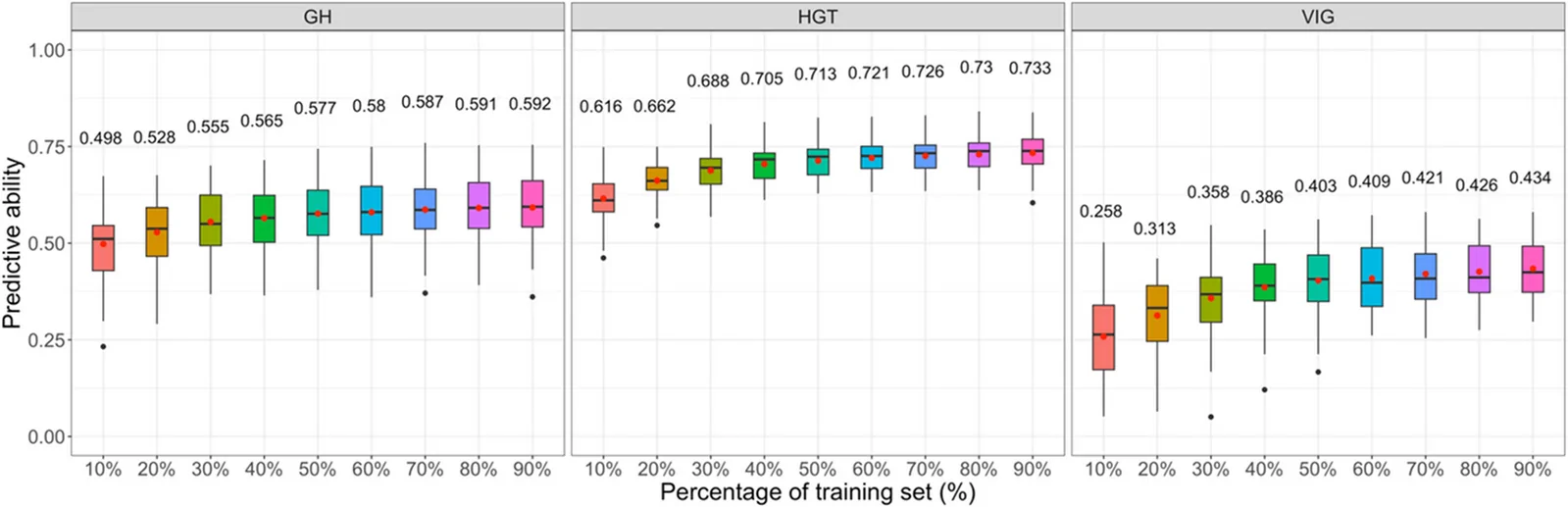
Predictive abilities for traits by genomic prediction with increasing equal sample proportions from each of the five subgroups in the training set. Red dots represent each group’s mean predictive ability, with specific values displayed above the interquartile-range boxes

The effect of increasing equal sample proportions from each of five subgroups in the training set on genomic prediction abilities is shown in Fig. 4. A similar trend was observed across all traits, with predictive ability gradually increasing as the training set sample proportion increased from 10% to 90%. Among the traits, HGT consistently showed the highest predictive ability across all sample proportions, whereas VIG showed the lowest. At the 10% sample proportion, the average predictive abilities were the lowest for GH (0.498), HGT (0.616), and VIG (0.251). Maximal average predictive abilities were achieved at the 90% sample proportion, with values for GH (0.592), HGT (0.733), and VIG (0.434), representing increases of 19% for both GH and HGT and 73% for VIG compared to the 10% proportion. Predictive abilities of both GH and HGT increased slowly with each 10% increment in sample proportion (Supplemental Table S5). As the sample proportion increased, the increase in predictive ability declined from 6% (at 20%) to 0% (at 90%) for GH, and from 8% (at 20%) to 1% (at 90%) for HGT. Additionally, VIG showed larger percentage increases at lower sample proportions (25% from 10% to 20%, 14% from 20% to 30%), but the rate of increase slowed at higher proportions.

### Assessment of genomic prediction performance with increasing samples from training subgroups versus the test subgroup to the training set

**Fig. 5:**
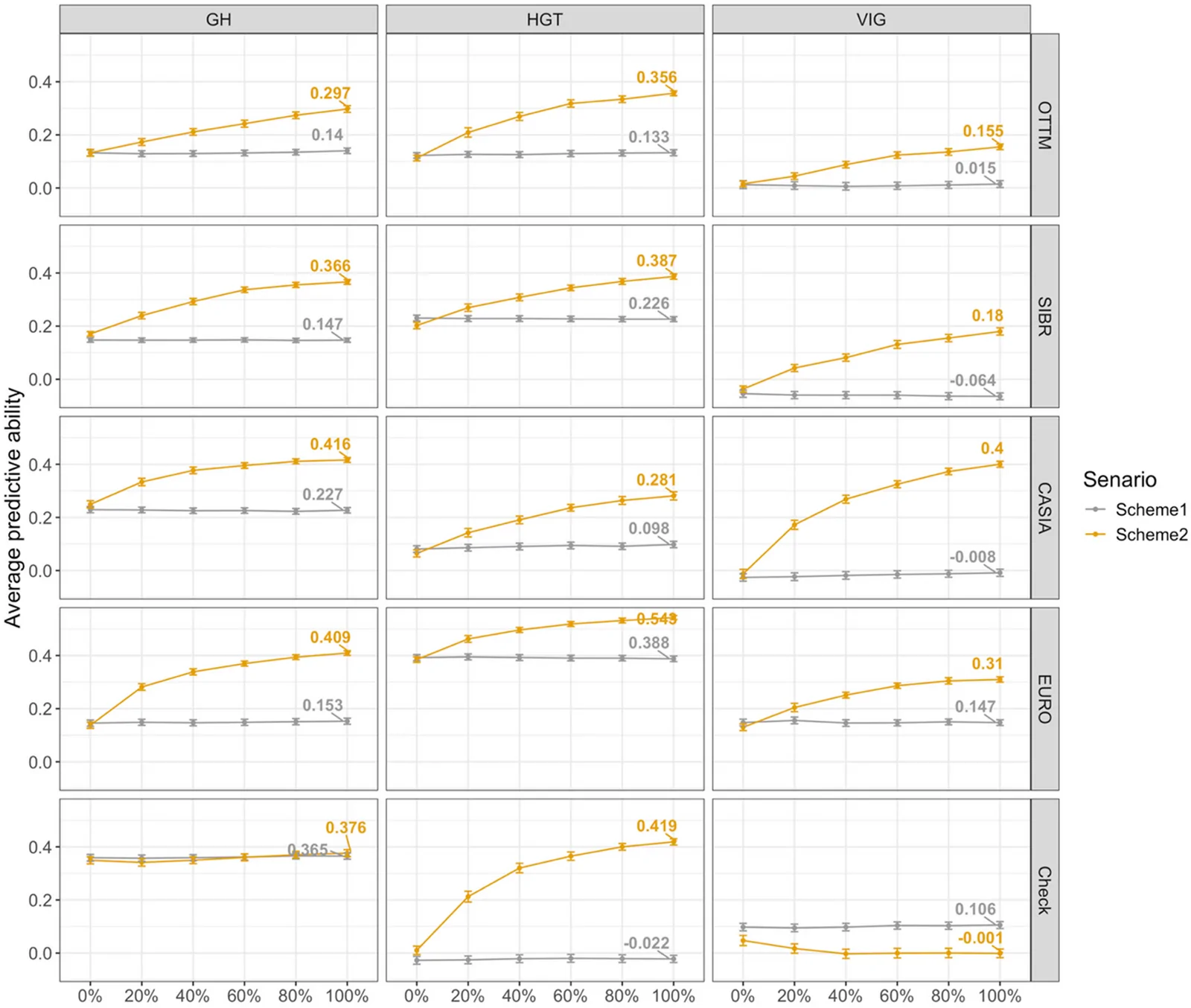
Average predictive abilities for traits in each subgroup by genomic prediction with increasing proportions of samples (0%~100%, in 20% increments) to the training set: comparison between Scheme1 and Scheme2. Scheme1: The excluded samples from the other four training subgroups were added to the training set with an increasing proportion (0%–100%, in 20% increments). Scheme2: The remaining 50% of the test subgroup, equal in size to the excluded samples in Scheme1, was added to the training set with an increasing proportion (0%–100%, in 20% increments)

Predictive abilities for the three traits varied among subgroups between the two prediction schemes (Fig. 5; Supplemental Table S6). In Scheme1, test subgroup samples were not included in the training set; similar average predictive abilities were observed across all traits and subgroups despite increasing the proportion of additional training samples (0%–100%, in 20% increments) excluded from other four training subgroups to the training set, which was supported by the genetic relatedness between the test and training sets that did not show obvious increase across proportions (Supplemental Figure S6; Supplemental Table S7). In contrast, in Scheme2, the incorporation of the remaining test subgroup samples (0%–100%, in 20% increments) matched in size to the corresponding proportions of additional training samples (in Scheme1) to the training set showed that average predictive abilities were gradually improved compared to Scheme1 (Fig. 5), which was also accompanied by a gradual increase in the genetic relatedness (Supplemental Figure S6; Supplemental Table S7), except for VIG in the Check subgroup (Fig. 5; Supplemental Figure S6). The best prediction performance in Scheme2 was achieved when 100% of the remaining test subgroup samples was included in the training set. At the 100% proportion, GH showed > 83% increase (except 3% in Check subgroup), HGT improved > 40%, and VIG increased > 100% (except in Check subgroup) (Supplemental Table S8). Predictive abilities ranged from 0.297 (OTTM) to 0.416 (CASIA) for GH, 0.281 (CASIA) to 0.543 (EURO) for HGT, 0.106 (Check) to 0.4 (CASIA) for VIG (Fig. 5).

### Assessment of genomic prediction performance for plant vigor (VIG) across different years

To mimic practical breeding scenarios in which data from earlier years are used to predict performance in subsequent years, we evaluated a series of cross-year prediction schemes (Supplemental Figure S7). When the 2016 dataset was used as the training set, predictive ability was higher for 2017 (0.360) than for 2018 (0.209). In contrast, using the 2017 dataset to predict performance in 2018 resulted in an improved average predictive ability of 0.286. Combining data from 2016 to 2017 to estimate BLUEs yielded a predictive ability of 0.270 for 2018, which was comparable to using the 2017 dataset alone. Overall, predictive ability was higher between adjacent years (2016 to 2017 and 2017 to 2018), whereas prediction from 2016 to 2018 was substantially weaker, and the inclusion of non-adjacent historical data might not further improve prediction beyond the most recent year.

A similar pattern was also observed at the level of SNP marker effects across years. To further investigate the underlying pattern of predictive ability, we evaluated the stability of SNP marker effects across years. Pearson correlations of marker effects (Supplemental Figure S8) showed the highest similarity between adjacent years, with stronger agreement between 2016 and 2017 (*r* = 0.706) and between 2017 and 2018 (*r* = 0.626), whereas the correlation between non-adjacent years 2016 and 2018 was substantially lower (*r* = 0.378). The combined 2016–2017 dataset showed intermediate similarity with 2018 (*r* = 0.571).

## Discussion

Genomic prediction (GP) has been widely adopted in plant breeding programs to accelerate the identification and selection of superior genotypes [49, 50]. The continued reduction in genotyping costs has further facilitated the integration of GP into diverse breeding strategies, enabling more efficient use of genetic resources [51, 52]. In this study, multiple GP schemes using the alfalfa DArTag genotyping platform were designed to represent realistic selection scenarios in alfalfa breeding, with particular emphasis on incorporating exotic germplasm. These schemes aimed to evaluate the impact of population structure, population composition, genetic relatedness between the training and test sets, and training set size on predictive ability, thereby providing insights into the development of effective GP workflows for alfalfa improvement. Modeling different combinations of geographic backgrounds highlighted the importance of strategic training-population design to optimize predictive performance, especially when introducing highly diverse germplasm into breeding pipelines.

In this study, a PCA based on genotype profiles revealed population grouping patterns that were largely consistent with the geographic origins of each subgroup. The PCA plot indicated that the CASIA, EURO, and SIBR subgroups exhibited greater genetic diversity than the other subgroups, as evidenced by their broader dispersion. Partial overlap was also observed among the EURO, OTTM, and Check groups, suggesting shared genetic backgrounds or gene flow among these populations. A few studies have reported PCA analyses using different alfalfa germplasm from these geographic regions. A previous study by Pégard et al. (2023) investigated plant materials from seven geographic groups, including China, North Europe, and North America, which partially overlap with our geographic subgroups, CASIA, EURO, and Check, respectively [53]. Although discriminant analysis of principal components (DAPC) is not directly comparable to PCA, both analyses revealed two consistent patterns: (1) accessions from China and CASIA appear to be more distinct from North American accessions, and (2) European and North American samples exhibit relatively close genetic relationships. Because American germplasm likely originated from a single ancestral population in Europe [54], the observed overlap of European and American genetic diversity was expected.

The pairwise genetic differentiation coefficient (*F*_*ST*_) and Nei’s genetic distance (*D*_*A*_) estimates indicated that genetic divergence existed among these subgroups but remained low. Similarly, previous studies in alfalfa have reported low but significant *F*_*ST*_ values, ranging from 0 to 0.012 based on SSR markers [55], from 0.003 to 0.028 based on SSR markers [56], and from 0.001 to 0.026 based on SNP markers [53]. Despite the relatively low *F*_*ST*_ and *D*_*A*_ estimates, different linkage disequilibrium (LD) block sizes were observed across the entire population and within each of the five subgroups. At *r*^*2*^ = 0.1, LD decay distance was approximately 0.50 Mb in the entire population (All), while subgroup-specific LD decay was slower, with estimated distances ranging from 1.02 Mb in OTTM, 1.47 Mb in Check, 1.70 Mb in EURO, 2.12 Mb in CASIA, to 2.36 Mb in SIBR. LD decay distance estimates may not be directly comparable across studies, as they are influenced by differences in population structure, marker density, and sample size. In addition, different thresholds can yield different estimates of LD decay distance, with *r²* = 0.1 and *r²* = 0.2 being the most used cutoffs in LD decay analyses. Li et al. (2014) reported that the extent of LD rapidly decayed to 26 kb at *r*^*2*^ = 0.2 across 280 alfalfa genotypes, including related species, but that it varied among populations [57]. Murad Leite Andrade et al. (2022) observed an LD decay distance of approximately 433 kb at *r*^*2*^ = 0.2 in a diverse set of parental lines from 33 populations from various germplasm sources [58]. Notably, a previous study by Medina et al. (2025) analyzed the same germplasm with the same 3 K DArTag panel used here, with only some differences in the Check subgroup, but twice the sample size compared to our study [20]. The study also reported that different linkage disequilibrium (LD) block sizes were observed across the entire population and within each subgroup [20], albeit with lower LD decay distances (110 kb~181 kb) than our results at *r*^*2*^ = 0.1; such discrepancies are likely due to differences in sample size [59, 60].

Predictive abilities of GP can be influenced by multiple factors, including the genetic architecture of the trait, linkage disequilibrium, population structure, population composition, genetic relatedness between the training and test sets, and the size of the training set [61–64]. Predictive ability can therefore vary considerably depending on the underlying genetic architecture of the trait. Previous studies have reported high predictive abilities for qualitative traits, such as anthracnose resistance [53], plant height and fall dormancy [53], creeping rootedness [21], dry matter yield in biomass yield [7], and low predictive abilities for highly quantitative traits, like biomass (Annicchiarico et al. 2015) and dry matter yield [19, 58]. Limited phenotypic variation can reduce predictive ability. In this study, we observed low predictive ability of vigor (VIG) (*r* = 0.008) in the Check subgroup in the within-subgroup five-fold cross-validation analysis. This subgroup consisted of U.S. commercial cultivars (‘55H94’, ‘Genoa’, ‘Hybriforce-4400’, and ‘Vernal’) selected to represent high-yielding elite germplasm adapted to U.S. growing conditions. Notably, the Check subgroup exhibited the highest mean VIG value (5.3) and the lowest coefficient of variation (CV = 26.7%) among all subgroups (Table 1), indicating limited phenotypic variation. The narrow phenotypic distribution reflects prior intensive selection within elite breeding germplasm. Consequently, the combination of high mean performance and low phenotypic variability may have reduced the ability to accurately estimate genetic effects at associated loci, leading to the low predictive ability observed in the Check subgroup.

Predictive ability can also be influenced by linkage disequilibrium (LD). Species with rapid LD decay generally require more markers associated with target traits to achieve greater predictive power. However, increasing marker density does not always lead to proportional improvements in prediction accuracy. Sipowicz et al. (2025) evaluated the effect of marker density on genomic prediction in alfalfa by comparing DArTag marker sets ranging from 10 to 3,000 markers with Capture-seq marker sets ranging from 10 to 11,000 markers [19]. They found that predictive ability increased with marker density until it plateaued at approximately 1,000 markers. Therefore, the 3 K alfalfa DArTag marker set used in this study likely represents a cost-effective and sufficient marker density for genomic prediction in alfalfa.

Population structure also plays an important role in determining GP performance. Within-subgroup GP was performed under reduced population structure, whereas GP across the entire population (five subgroups) involved stronger population structure in this study. Predictive ability increased substantially in the entire population compared to those within subgroups, representing improvements of 45% (EURO) to 109% (Check) for HGT, 36% (EURO) to 112% (CASIA) for GH, and > 36% for VIG except in CASIA but remained comparable **(**Fig. 3**)**. In populations exhibiting moderate to high population structure, predictive ability also can often be improved by implementing stratified sampling compared with completely random sampling in the entire population [65]. A similar trend was observed in this study. Under the stratified sampling scheme, predictive abilities reached 0.730 for HGT and 0.426 for VIG when 80% of the entire population was included in the training set (Fig. 4), representing slight improvements over the random sampling scheme applied to the entire population (0.725 for HGT and 0.412 for VIG) (Fig. 3). Predictive ability for GH was slightly lower under the stratified sampling scheme (0.592) but remained comparable to that obtained with random sampling (0.603).

Previous studies have also reported that predictive ability tends to decline when the training population is genetically less related to the test population [66]. Our findings are consistent with this observation, as training populations derived from certain geographic regions showed limited predictive ability for germplasm originating from a new geographic region (Fig. 5, Scheme1). This result suggests that when genetically distinct materials are introduced, prediction models trained exclusively on the original germplasm may have limited transferability. However, incorporating even a subset of materials from the new geographic region that served as the test group into the training population substantially improved predictive ability (Fig. 5, Scheme2). The rate of improvement in predictive ability was greatest when a small batch of samples from the new geographic region was added, whereas the rate of increase slowed once the new germplasm in the training set reached approximately 80%–100% of the test population size (Fig. 5, Scheme2). This finding suggests that breeders may achieve reasonable predictive abilities with relatively limited additional investment by phenotyping only a subset of germplasm from the new geographic region to update the prediction model.

It is widely recognized that increasing training population size can improve predictive ability. The predictive ability increased gradually for all traits as the training set sample proportion increased from 10% to 90% under the stratified sampling scheme in the entire population (Fig. 4), and thereafter it plateaued (Supplemental Table S5). Wang et al. (2024) similarly observed that predictive ability slightly increased as the size of the training population increased in alfalfa [67]. Similarly, previous studies in wheat [66] and soybean [68] also observed that prediction ability improves with increasing training set size and reaches a plateau. However, when additional samples from a distinctive genetic region that served as the test group are incorporated into the training set, the observed changes in predictive ability may reflect not only the increase in sample size but also concurrent alterations in the genetic relatedness between the training and test sets. To isolate the effect of sample size, we designed Scheme1 by adding samples with different proportions from the original geographic regions to the training set (Fig. 2; Scheme1). The absence of a corresponding increase in predictive ability under this design suggests that enhanced genetic relatedness, rather than increased sample size alone, was the primary factor driving the improvement in predictive ability (Fig. 5; Scheme1), supported by the previous study [68]. To further test this hypothesis, genetic relatedness between the training and test sets was evaluated as additional samples from the new geographic region that served as the test group were incorporated into the training set (Fig. 2; Scheme2). As expected, predictive ability increased as genetic relatedness between the test and training sets became stronger (Supplemental Figure S6). Although we did not evaluate optimized training population approaches such as the CDmean method [69], we anticipate that comparable predictive abilities for germplasm from new geographic regions or genetic clusters could be achieved using smaller, strategically selected training populations. These findings suggest that breeders may be able to update genomic prediction models for newly introduced germplasm more efficiently, particularly in breeding programs where phenotypic evaluation is costly.

Various modeling approaches have been explored for genomic prediction in plant breeding. In addition to the widely used rrBLUP model, alternative methods have been developed to capture complex, non-linear genetic architectures and non-additive interactions (epistasis). These models, including but not limited to Bayesian approaches (e.g., BayesA, BayesB, Bayesian LASSO) that allow different assumptions regarding marker effect distributions, machine learning approaches (e.g., Random Forest, Extreme Gradient Boosting) that can accommodate complex interactions between markers, have demonstrated superior predictive performance for traits influenced by non-additive genetic effects because they can effectively model non-linear relationships [70–74]. However, non-additive effects depend on specific combinations of alleles and/or loci, which may be disrupted during meiosis, thereby limiting their transferability across generations [73]. Therefore, this study focused on evaluating different prediction schemes using the rrBLUP model, which efficiently captures additive genetic effects and has consistently demonstrated robust performance in genomic prediction studies [48]. In addition, additive genetic effects remain the primary driver of long-term genetic gain because they represent the heritable component that is reliably transmitted across generations [75, 76].

Genotype-by-environment (G×E) interaction remains a major challenge in plant breeding. Environmental covariates such as photoperiod, temperature, precipitation, soil moisture, and growing degree days have been extensively monitored and incorporated into predictive models to improve predictions of environment-specific plant performance in breeding programs [77–79]. Alternatively, multi-environment GBLUP models incorporate environmental kernels to characterize similarities among environments and borrow information across environments for genomic prediction [77]. In prediction scenarios, the main genomic breeding values for GH, HGT, and VIG were modeled using BLUEs estimated across years. Although the use of BLUEs instead of best linear unbiased predictions (BLUPs) as phenotypic inputs may influence genomic prediction ability estimates under certain experimental conditions (e.g., unbalanced datasets or experimental designs with limited replication), and then explicit comparisons between BLUE- and BLUP-based phenotypic inputs to assess potential impact on predictive ability could help determine the most appropriate input for GP, the phenotypic values in this study were estimated as BLUEs using data collected across multiple years or multiple cuts per year, which can be considered as balanced dataset. Therefore, the potential impact of using BLUEs instead of BLUPs is likely to be limited in this study. However, G×E interactions could not be explicitly estimated because true replications within each cut were unavailable. Consequently, the reported predictive abilities from prediction scenarios reflect the average performance of the evaluated prediction models across environments but may be inflated for a specific environment. In the temporal prediction schemes, BLUEs for VIG were estimated separately for each year, thereby capturing both general and environment-specific genetic effects. As a result, prediction models trained using BLUEs from 2016 showed lower predictive ability when predicting performance in 2018 than in 2017, and lower than models trained using 2017 to predict 2018, likely due to G×E interactions, which were comparable with the previous genomic prediction study in loblolly pine [80]. These environment-specific genetic effects were further reflected in the correlations of marker effects estimated in different years using the rrBLUP model (Supplementary Figure S8). Marker effect estimates showed substantially higher stability between adjacent years (*r* = 0.706 for 2016 vs. 2017; *r* = 0.626 for 2017 vs. 2018) than between non-adjacent years (*r* = 0.378 for 2016 vs. 2018). This decline in predictive ability and marker-effect stability with increasing temporal distance suggested that the genetic control of VIG is not fully stable across years, likely reflecting G×E interactions that may alter QTL expression [64, 81]. In future studies, incorporating G×E interactions more effectively in the temporal prediction schemes could further improve predictive ability and enhance the robustness and transferability of prediction models across environments. Notably, alfalfa is a perennial forage crop, and plant vigor is strongly correlated across years. In line with this expectation for VIG, strong positive phenotypic correlations were also observed among adjacent years (2016 vs. 2017; 2017 vs. 2018), whereas weaker correlations were detected for more temporally distant comparisons (2016 vs. 2018) (Supplemental Figure S2). In the across-year prediction analyses for VIG, we observed that data from 2016 produced the highest predictive ability for predicting performance in 2017 (Supplementary Figure S7). However, because the study included environments from only three consecutive years, we were unable to disentangle changes in plant status over time from true G×E interactions.

## Conclusions

This study provides insights into effective genomic prediction strategies for incorporating novel genetic diversity into alfalfa breeding programs in North America. Using a highly diverse germplasm panel with moderate population structure representing five geographic origins, we demonstrated that stratified sampling across all subgroups generally resulted in higher predictive ability than either within-subgroup sampling or slightly higher than completely random sampling from the entire population. Our results further showed that increasing the training population size under the stratified sampling scheme improved predictive ability. However, when introducing germplasm from the new geographic region that served as the test group to the training set, increased genetic relatedness between the training and test sets could be a major driver of improved predictive performance. Temporal prediction and maker effect analyses also suggested that G×E interactions are critical for improving the robustness and transferability of prediction models across environments. Overall, this study reflects practical breeding scenarios encountered in alfalfa breeding programs and provides an evaluation of prediction schemes that may be suited to different breeding objectives.

## Supplementary Information

Supplementary Material 1: Supplemental Figure S1. Mowing dates for the plant materials and phenotyping dates for each trait. Supplemental Figure S2. Correlation coefficient matrix, scatter plot, and phenotypic frequency distributions of phenotypic traits. The diagonal shows the distribution of best linear unbiased estimates (BLUEs) for each trait. GH: Plant growth habit; HGT: Plant height; VIG: Plant vigor. GH, VIG_2016, VIG_2017, and VIG_2018 represent BLUEs estimated across all cuts within a year. HGT, VIG_2016&17, and VIG represent the BLUEs estimated across several years. Below the diagonal are bivariate scatter plots with fitted linear regression lines and 95% confidence intervals. Above the diagonal are Pearson correlation coefficients, with significance levels indicated by stars: *** for p < 0.001, ** for p < 0.01, and * for p < 0.05. Supplemental Figure S3. Principal component analysis (PCA) of five subgroups (OTTM, SIBR, CASIA, EURO, and Check) based on 3,756 SNPs. Supplemental Figure S4. Linkage disequilibrium (LD) decay curve calculated through GWASpoly software for the entire population (All) as well as each subgroup using the 3,756 SNPs. Linkage block size was determined as the chromosomal distance where r2 =0.1. Supplemental Figure S5. Heatmap of Nei’s genetic distance (DA, upper diagonal) and pairwise genetic differentiation (FST, lower diagonal) between pairs of subgroups. Supplemental Figure S6. Plot of the relationship between average predictive ability and genetic relatedness between the test and training sets across increasing training sample proportions (0%–100%, in 20% increments). For each proportion, the corresponding genetic relatedness was used as the x-axis value, and the average predictive ability was used as the y-axis value. Each point represents a specific proportion under the two schemes. Scheme1: The excluded samples from the other four training subgroups (G) were added to the training set with increasing proportions (0%–100%, in 20% increments). Scheme2: The remaining 50% of the test subgroup, equal in size to the excluded samples in Scheme1, was added to the training set with increasing proportions (0%–100%, in 20% increments). Supplemental Figure S7. Boxplot of genomic predictive ability for plant vigor (VIG) across different years. Four cross-year prediction schemes were evaluated: training using BLUEs from 2016 to predict 2017 or 2018; training in 2017 to predict 2018; and training on the combined experiment from 2016 and 2017 to predict 2018. Red text above each box represents the mean of predictive abilities of 50 iterations of five-fold cross-validation, where predictive ability is calculated as Pearson’s correlation between the BLUEs and predicted values. Supplemental Figure S8. Pairwise correlations of SNP marker effects estimated across years and combined datasets for VIG. Scatter plots of SNP marker effects (β) between 2016, 2017, 2018, and the combined 2016–2017 dataset. Pearson correlation coefficients (r) are shown in each panel. Each point represents a SNP.

Supplementary Material 2: Supplemental Table S1. Plant Introduction (PI) for polycross to produce four experimental germplasms (CASIA, EURO, OTTM, SIBR). Supplemental Table S2. Bayesian Information Criterion (BIC) and Akaike Information Criterion (AIC) values for choosing the best model to calculate the best linear unbiased estimates (BLUEs). Supplemental Table S3. Best linear unbiased estimates (BLUEs) for each trait using the best model. Supplemental Table S4. Predictive abilities within each of the five subgroups versus across the entire population (All), with fifty iterations for each trait in each subgroup. Supplemental Table S5. Average predictive abilities for traits by genomic prediction with increasing equal sample proportions from each of the five subgroups in the training set. Supplemental Table S6. Average predictive abilities (Mean), and standard errors (SD) for traits in each subgroup using Scheme1 and Scheme2. Supplemental Table S7. Genetic relatedness and predictive abilities at each proportion (0%–100%, in 20% increments) under Scheme1 and Scheme 2, with fifty iterations for each trait in each subgroup. Supplemental Table S8. Average predictive abilities at 100% proportion using Scheme1 and Scheme 2 for each trait in each subgroup.

## Data Availability

The code and data are available in our GitHub repository (https://github.com/Breeding-Insight/Alfalfa_genomic_prediction_paper).

## Abbreviations

AIC: Akaike information criteria
Alt: Alternative allele
AltMatch: Alternative allele match with other variant loci
AOSCA: Association of Official Seed Certification Agencies
AR: Autoregressive
BIC: Bayesian information criteria
BLAST: Basic local alignment search tool
BLUE: Best linear unbiased estimation
CASIA: Central Asia
cm: Centimeters
CV: Coefficient of variation
DArT: Diversity Arrays Technology
DArTag: Diversity Arrays Technology targeted genotyping platform
EURO: Europe
G: Germplasm group
GBLUP: Genetic best linear unbiased prediction
GH: Plant growth habit
GP: Genomic prediction
GWAS: Genome-wide association study
GxE: Genotype-by-environment
h: Narrow-sense heritability
HGT: Plant height
K: Thousands
LD: Linkage disequilibrium
MADC: Missing allele discovery counts
MAF: Minor allele frequency
Mb: Mega base pairs
NCBI: National Center for Biotechnology Information
NPGS: National Plant Germplasm System
OTTM: Ottoman
PC: Principal component
PCA: Principal component analysis
PEV: Prediction-error variance
PI: Plant introduction
QTL: Quantitative trait locus
Ref: Reference allele
RefMatch: Reference allele match with other variant loci
rrBLUP: Ridge-regression best linear unbiased prediction
SD: Standard deviation
SIBR: Siberia/Mongolia
SNP: Single-nucleotide polymorphism
SSR: Simple sequence repeat
U.S.: United States
VIG: Plant vigor
