# Supplementary material for: Enhancing alfalfa breeding through genomic prediction with exotic germplasm resources
Source: BMC Plant Biol. 2026 Jul 18;26:1757. doi: 10.1186/s12870-026-09507-5 (PMC13660186; doi:10.1186/s12870-026-09507-5)
Supplement: Supplementary file 1 [file 12870_2026_9507_MOESM1_ESM.docx]

**Supplemental Figure S1** Mowing dates for the plant materials and phenotyping dates for each trait.

**Supplemental Figure S2** Correlation coefficient matrix, scatter plot, and phenotypic frequency distributions of phenotypic traits. The diagonal shows the distribution of best linear unbiased estimates (BLUEs) for each trait. GH: Plant growth habit; HGT: Plant height; VIG: Plant vigor. GH, VIG_2016, VIG_2017, and VIG_2018 represent BLUEs estimated across all cuts within a year. HGT, VIG_2016&17, VIG represent the BLUEs estimated across several years. Below the diagonal are bivariate scatter plots with fitted linear regression lines and 95% confidence intervals. Above the diagonal are Pearson correlation coefficients, with significance levels indicated by stars: *** for p < 0.001, ** for p < 0.01, and * for p < 0.05.

**Supplemental Figure S3** Principal component analysis (PCA) of five subgroups (OTTM, SIBR, CASIA, EURO, and Check) based on 3,756 SNPs.

**Supplemental Figure S4** Linkage disequilibrium (LD) decay curve calculated through GWASpoly software for the entire population (All) as well as each subgroup using the 3,756 SNPs. Linkage block size was determined as the chromosomal distance where *r^2^* =0.1.

**Supplemental Figure S5** Heatmap of Nei’s genetic distance (DA, upper diagonal) and pairwise genetic differentiation (FST, lower diagonal) between pairs of subgroups.

**Supplemental Figure S6** Plot of relationship between average predictive ability and genetic relatedness between the test and training sets across increasing training sample proportions (0%–100%, in 20% increments). For each proportion, the corresponding genetic relatedness was used as the x-axis value, and the average predictive ability was used as the y-axis value. Each point represents a specific proportion under the two schemes. Scheme 1: the excluded samples from the other four training subgroups were added to the training set with increasing proportions (0%–100%, in 20% increments). Scheme 2: the remaining 50% of the test subgroup, equal in size to the excluded samples in Scheme1, was added to the training set with increasing proportions (0%–100%, in 20% increments).

c

**Supplemental Figure S7** Boxplot of genomic predictive ability for plant vigor (VIG) across different years. Four cross-year prediction schemes were evaluated: training using BLUEs of 2016 to predict 2017 or 2018, training in 2017 to predict 2018, and training on combined experiment of 2016 and 2017 to predict 2018. Red text above each box represents the mean of predictive abilities of 50 iterations of five-fold cross validation, where predictive ability is calculated as the Pearson’s correlation between the BLUEs and predicted values.

**Supplemental Figure S8** Pairwise correlations of SNP marker effects estimated across years and combined datasets for VIG. Scatter plots of SNP marker effects (β) between 2016, 2017, 2018, and the combined 2016–2017 dataset. Pearson correlation coefficients (*r*) are shown in each panel. Each point represents a SNP.
